# Prematurity and biliary atresia: a 30-year observational study

**DOI:** 10.1007/s00383-017-4193-1

**Published:** 2017-10-13

**Authors:** Natalie Durkin, Maesha Deheragoda, Mark Davenport

**Affiliations:** 1 Department of Paediatric Surgery, King’s College Hospital, London, SE5 9RS UK; 2 Institute of Liver Studies, King’s College Hospital, London, SE5 9RS UK

**Keywords:** Biliary atresia, Twins, Prematurity, Liver fibrosis, Outcome

## Abstract

**Aim of study:**

The diagnosis of biliary atresia (BA) remains challenging and delay can lead to significant morbidity with time to surgery a key factor in determining outcome. Prematurity may impact on outcome potentially delaying diagnosis. We sought to assess whether the premature BA infants (PBA) have a delayed time to surgery and as such, worse outcomes?

**Methods:**

Review of a single-centre prospectively maintained database. Prematurity was defined as delivery < 37/40 gestation. PBA was compared with date-matched term biliary atresia controls on a 2:1 basis. Primary outcomes were clearance of jaundice (< 20 μmol/L) and native liver survival. A retrospective assessment of liver fibrosis was made on biopsies at diagnosis and at Kasai portoenterostomy (KPE) in both premature and term cohorts. Data are quoted as median (range) unless indicated. A *P* value of ≤ 0.05 was considered statistically significant.

**Results:**

21 (female *n* = 14, 67%) premature infants with BA were treated in the period Jan. 1988–Dec. 2016 and compared with 41 contemporaneous term BA controls. Median gestation was 33 (29–36) weeks and birth weight 1930 (948–4230)g. Twin pregnancy (*n* = 10) was the leading cause for prematurity and significantly higher than the controls (48 vs. 0%; *P* < 0.0001). Maternal co-morbidity was high (*n* = 10, 48%) including pre-eclampsia (19%) and diabetes (14%). Liver biopsy was performed in 19 (90%) patients (all diagnostic) at a median of 57 (4–266) days. Delayed diagnosis (> 50 days) was seen in *n* = 13 but not associated with parenteral nutrition use (46 vs. 33%, *P* = 0.59) or phototherapy (50 vs. 83%, *P* = 0.19). Both BASM (33 vs. 7.5%; *P* = 0.01) and duodenal atresia (19 vs. 0%; *P* = 0.01) were seen more frequently in the PBA cohort. Mean fibrosis scores (Ishak) from diagnostic biopsies were lower in the premature group than the control group (2.71 vs. 3.53, *P* = 0.043) indicating less fibrosis but this equalized by time of subsequent KPE (*P* = 0.17). Primary surgery was Kasai portoenterostomy (*n* = 20) at an older median age than controls (65 vs. 56 days; *P* = 0.06). Liver transplantation was the primary procedure in one late-presenting child. There was an increased but non-significant clearance of jaundice in the PBA group [*n* = 12/20 (60%) vs 20/41 (48%); *P* = 0.23] post-KPE. Native liver survival and true survival were not different (*P* = 0.58 and 0.23).

**Conclusions:**

PBA infants have similar outcomes to term infants, despite delayed diagnosis and higher frequency of the syndromic form. The high incidence of discordant twins supports the theory that epigenetic modifications could contribute to the pathogenesis of BA.

**Level of evidence:**

IIIc Retrospective Matched Cohort Study.

## Introduction

Biliary atresia is an obstructive cholangiopathy of essentially unknown aetiology which, left untreated, is a potent cause of liver fibrosis and ultimately secondary biliary cirrhosis [1]. Possible causes include a developmental disorder of the bile ducts either in isolation or in association with other anomalies [2], bile duct ischaemia, or acquired bile duct injury secondary to a viral-mediated or triggered immune response [3, 4]. The onset of the injury may vary from early in the first trimester during embryonic organogenesis (e.g., Biliary Atresia Splenic Malformation (BASM)) to perhaps the perinatal period (e.g., CMV IgM+ve associated BA). Much of the timing of the latter is, however, based on speculation derived from various viral-induced animal models of BA [5–8] rather than evidence in the human condition.

Typically, infants with BA are born at term gestation with a normal birth weight; and interestingly this is true for those with either isolated BA or those of embryonic origin [9]. Recent evidence from incidental blood sampling on day 1 or day 2 of life also suggest that most if not all have a demonstrable conjugated hyperbilirubinaemia which implies that at the time of birth there is already established biliary obstruction [10].

Makin et al. reported on three infants who underwent laparotomy within the first week of life for intestinal atresia who were noted to have a likely BASM malformation [11]. All three patients were observed to have macroscopically normal livers, a finding which was further confirmed on histology from intra-operative wedge resection. This is despite not having any bile flow during their entire intrauterine life because they had the syndromic form of BA.

The premature infant with BA (PBA) is rare, and this remains a remote cause of conjugated jaundice in this cohort. Nevertheless, it must occur, though for a multiplicity of reasons the diagnosis may be delayed. The aim of this study was twofold; to assess outcome in premature infants with BA and secondly to explore the hypothesis that liver fibrosis is indeed time dependent and begins perinatally irrespective of its putative cause.

## Methods

Retrospective review of a prospectively maintained database (1988 to 2016) at a single centre. Prematurity was defined as < 37/40 (259 post-conceptual days) gestation. To try and negate the effect of changes in practice over time (e.g., obstetric and adjuvant use of steroids, different surgeons), we used contemporaneous control term BA (CBA) infants on a 2:1 basis. The primary measure of outcome was clearance of jaundice (< 20 µmol/L). The secondary outcomes were native liver and overall survival. BASM syndrome was defined by the presence of typical anomalies including polysplenia/absent spleen, situs inversus, pre-duodenal portal vein, cardiac anomalies and the absence of vena cava [2].

Liver biochemistry at the time of diagnostic liver biopsy, referral or KPE was ascertained and an aspartate aminotransferase/platelet ratio (APRi) calculated as a surrogate marker of liver fibrosis [12]. Percutaneous liver biopsy has and continues to be the diagnostic investigation of choice in our institution prior to KPE. Where diagnostic doubt existed on table, a cholangiogram was used to confirm the diagnosis. Formalin-fixed liver biopsy material in the form of an initial needle liver biopsy and an intra-operative wedge specimen at the time of Kasai portoenterostomy have been stored since patient presentation. Hematoxylin and eosin, and Picrosirius Red stained slides were reviewed blindly by a single histopathologist with semi-quantification of liver fibrosis using a previously validated scoring method; the Ishak score (grades 0–6) [13].

Formal institutional ethical approval was not sought for this study as it was regarded primarily as an audit of outcome.

Data are quoted as median (range) unless otherwise specified. Fisher’s exact and Chi^2^ test were used for categorical variables. Univariate analysis was undertaken using unpaired T test or Mann–Whitney *U* test as appropriate. Correlation was assessed using Spearman-rank correlation. Native liver survival was illustrated by Kaplan–Meier survival curves and differences assessed using a log-rank test. A *P* value ≤ 0.05 was considered to be statistically significant.

## Results

692 infants were diagnosed with BA during the period January 1988–December 2016, of which 21 (3%) were premature. Of these, 67% were female (*n* = 14) with a median gestation of 33 (29–36) weeks and a birth weight of 1930 (948–4230) g. The baseline characteristics were compared with 42 (2:1) contemporaneous controls (Table 1).

**Table 1:** Clinical characteristics of PBA and control patients

Characteristic	Controls (*n* = 42)	Preterm (*n* = 21)	*P* value
Gender (% male)	38%	33%	0.78
Gestational age	Term	33 (29–36)	
Birth weight (kg)	3.1 (2.0–4.4)	1.93 (0.95–4.23^a^)	0.0001
Twin	0	10 (48%)	0.0001
Maternal co-morbidity	1 (2%)	10 (48%)	0.0001
BASM	3 (7.5%)	7 (33%)	0.011
Type 3 BA	42 (100%)	20 (95%)	0.33

^a^Macrosomic infant secondary to IDDM

### Demography

Median maternal age was 28.5 years (19–38) in the PBA cohort; however, maternal co-morbidity appeared high (*n* = 10, 48%) including pregnancy-induced hypertension (*n* = 6, 29%) and insulin-dependent diabetes (*n* = 3, 14%). Conversely, there was only a single case of co-morbidity in the CBA group [gestational diabetes, *n* = 1 (2%)]. Abnormal antenatal scans were seen in 38% (*n* = 8) of the pre-term group, although only one of these was consistent with (cystic) biliary atresia.

Twin pregnancy (*n* = 10) was the leading and obvious cause for prematurity in our cohort (48 vs. 0% *P* < 0.0001). Of the 671 term BA patients in the King’s series, there were only 5 non-premature twins. Other causes of prematurity included spontaneous rupture of membranes (*n* = 7, 33%), pre-eclampsia (*n* = 4, 19%) and placental abruption (*n* = 1, 5%). Antenatal steroids were administered in 5 (24%) pre-term infants (Table 1).

The majority of BA was type III in both groups (95% PBA vs. 100% control), with two infants having the cystic type 3 variant in either group. One patient born at 30-week gestation presented with a history of fluctuating jaundice and pale stools and was shown to have type II BA at 156 days which appeared to be more consistent with “acquired” BA [14]. Despite obvious cirrhosis, the child remains well and jaundice free following KPE.

BASM was seen significantly more frequently in the PBA cohort than in the CBA group (33 vs. 7.5%; *P* = 0.01). Interestingly, the premature singleton pregnancies were more likely to have BASM than twins (55 vs. 10%; *P* = 0.04). Concomitant duodenal atresia (DA) was seen in 4 PBA patients, which was significantly higher than in controls (19 vs. 0%; *P* = 0.01). Remarkably, of these 4 infants undergoing laparotomy for DA within the first few days of life, all subsequently diagnosed with BASM, only two underwent a liver biopsy at the initial laparotomy on days 4 and 11, respectively. Referral and liver biopsy was not performed until day 266 of life in one of these infants, as her persistent cholestatic jaundice was erroneously felt to be secondary to parenteral nutrition.

### Timing of operation and liver fibrosis

Percutaneous liver biopsy was performed in 19 (90%) of pre-term infants and 36 (88%) of controls and was highly suggestive of a diagnosis of BA in all patients where performed. Biopsies were performed later in PBA patients than controls post-natal age 57 (4–266) days vs. 47 (24–129) days (*P* = 0.17) (Table 2). Overall, over two-thirds of the premature patients (*n* = 13/19, 68%) had a delayed diagnosis, using post-natal age ≥ 50 days at biopsy as an arbitrary marker, with a very delayed diagnosis (≥ 100 days) in 4 (19%). This was compared with a diagnosis ≥ 50 post-natal days in only 47% (17/36) of the control group. Of the PBA patients diagnosed ≥ 50 post-natal days, there was a higher use of parenteral nutrition compared to those diagnosed < 50 days, although this was not statistically significant (50 vs. 29%; *P* = 0.3). Phototherapy use was not found to be associated with a delayed diagnosis and was actually used more frequently in those diagnosed earlier (85% <50 days vs. 45%, *P* = 0.15).

**Fig. 1:**
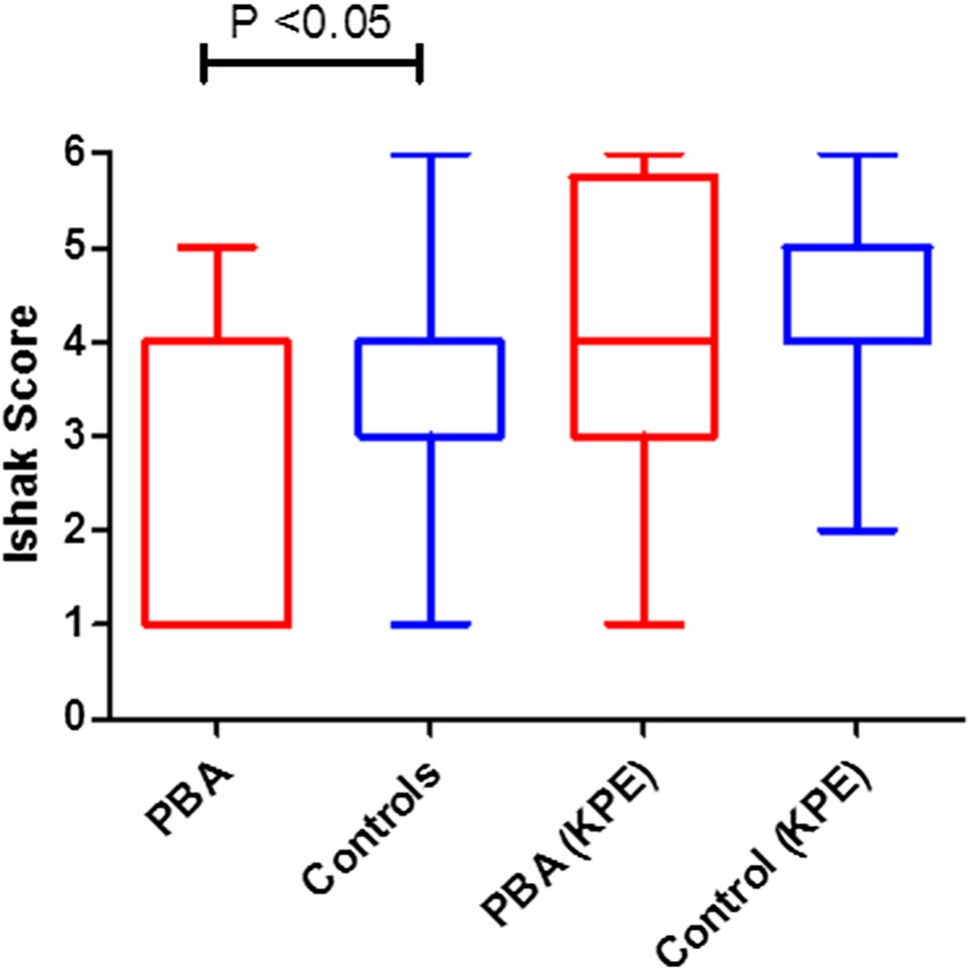
Mean fibrosis scores (Ishak) from diagnostic biopsy and at time of Kasai Portoenterostomy. Ishak scores are lower in the premature group than the control group (*P* = 0.04) at diagnosis but not at time of KPE (*P* = 0.17)

Despite later age at biopsy, mean Ishak fibrosis scores from diagnostic biopsies were actually lower in the premature group than the control group (2.71 vs. 3.53, *P* = 0.04) indicating less fibrosis at diagnosis. However this difference disappeared when fibrosis scores from the wedge biopsy at time of KPE were used (4.1 vs. 4.4; *P* = 0.17) (Fig. 1).

**Table 2:** Preoperative variables

	Term	Preterm	*P* value
Age at diagnostic biopsy (days)	47 (24–129)	57 (4–266)	0.17
Mean Ishak score (at diagnosis)	3.53 (± 1.30)	2.71 (± 1.69)	0.05
APRI at KPE	0.76	0.56	0.07
Bilirubin at KPE	170	129	0.0068
Age at Kasai (days)	56 (27–141)	65 (16–323)	0.03
Corrected age at KPE (weeks)	N/A	42 (36 + 5–70 + 2)	N/A
Mean Ishak score at KPE	4.48 (± 1.03)	4.13 (± 1.50)	0.33

**Fig. 2:**
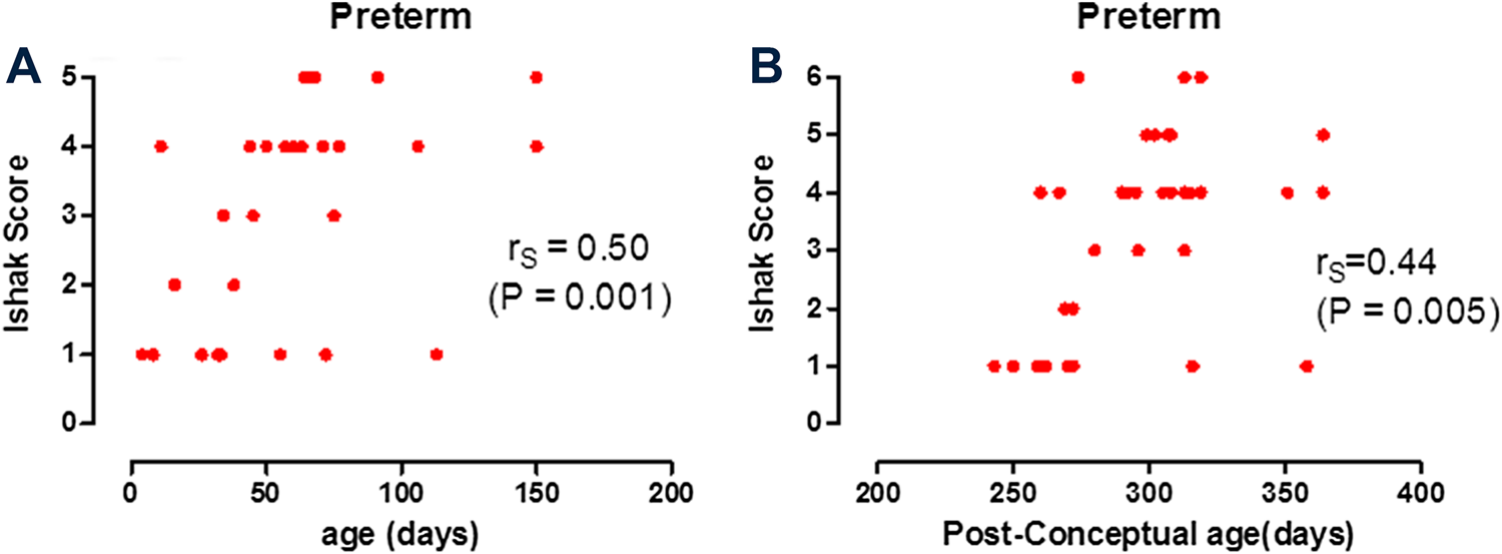
Liver fibrosis scores (Ishak) versus post-natal age (**a**) and post-conceptual age (**b**) in premature infants with biliary atresia. *N* = 33 samples

**Fig. 3:**
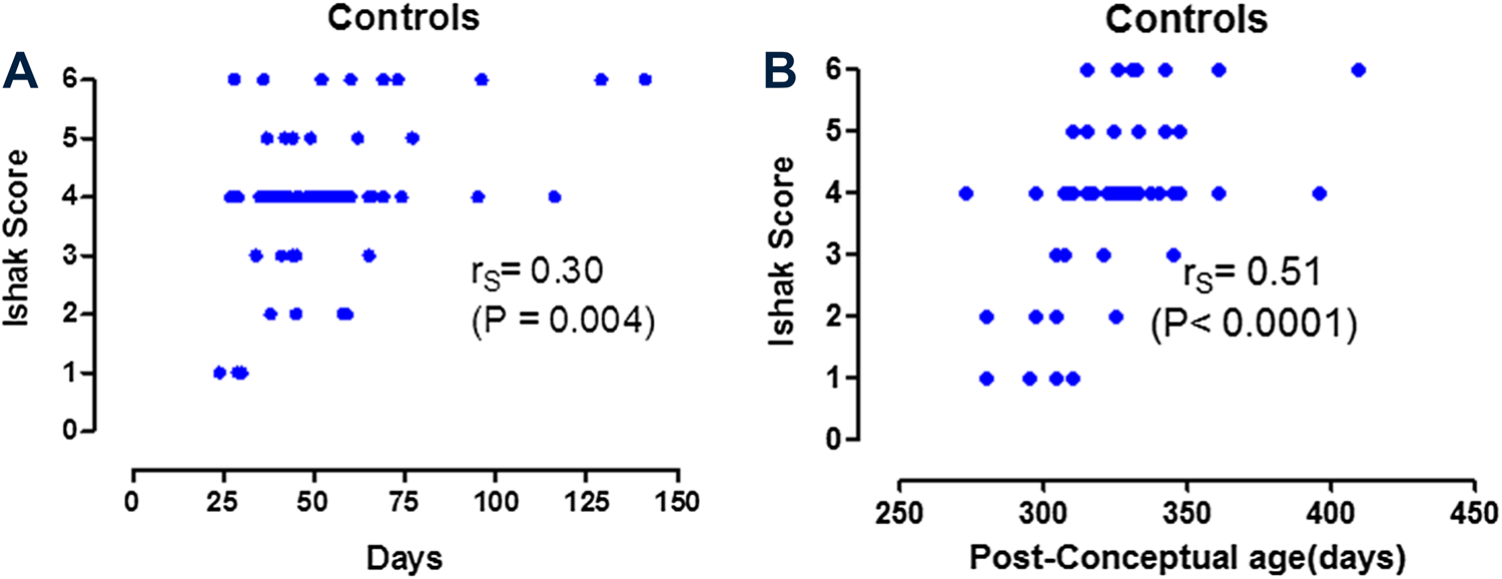
Liver fibrosis scores (Ishak) versus post-natal age (**a**) and post-conceptual age (**b**) in term infants with biliary atresia (controls). *N* = 66 samples

Figure 2a, b show the positive correlation between both post-natal age (*r*_S_ = 0.5; *P* = 0.001) and post-conceptual age (*r*_S_ = 0.44; *P* = 0.005) with liver fibrosis as assessed semi-quantitatively by the Ishak score for both the diagnostic and KPE biopsy in the premature group (*n* = 33). Figure 3a, b show this relationship between both post-natal age (*r*_S_ = 0.32; *P* = 0.004) and post-conceptual age (*r*_S_ = 0.51; *P* < 0.0001) for the control biopsy group (*n* = 66).

Kasai portoenterostomy (KPE) was the primary procedure in 20 (95%) PBA infants with primary transplantation in one infant who presented at 266 days of life (gestation 30 + 1, corrected age 197 days). Interestingly, her Ishak score was only 4 on wedge biopsy. All patients in the control group underwent KPE. As with age at diagnosis, median age at primary procedure was higher in the premature group (65 vs.56 days, *P* = 0.03). Despite being later to biopsy and operation, however, the median bilirubin was lower in the premature group at the time of operation (129 vs. 170, *P* = 0.007). Similarly, median APRi at the time of KPE was lower in the PBA cohort, but just failed to reach statistical significance [0.56 (0.11–5.2) vs. 0.72 (0.18–6.8); *P* = 0.07].

The deferral time (time from diagnosis with liver biopsy to operation) was also longer in our PBA group compared to controls (11 days vs. 7 days; *P* = 0.008), the reason for which is unclear. Of our five PBA patients operated at > 100 days, two presented from home with jaundice on days 83 and 91, two were reported to have ‘fluctuating jaundice’ with abnormal but not definitive ultrasound scans and one was thought to be secondary to PN (discussed earlier).

### Outcomes

**Fig. 4:**
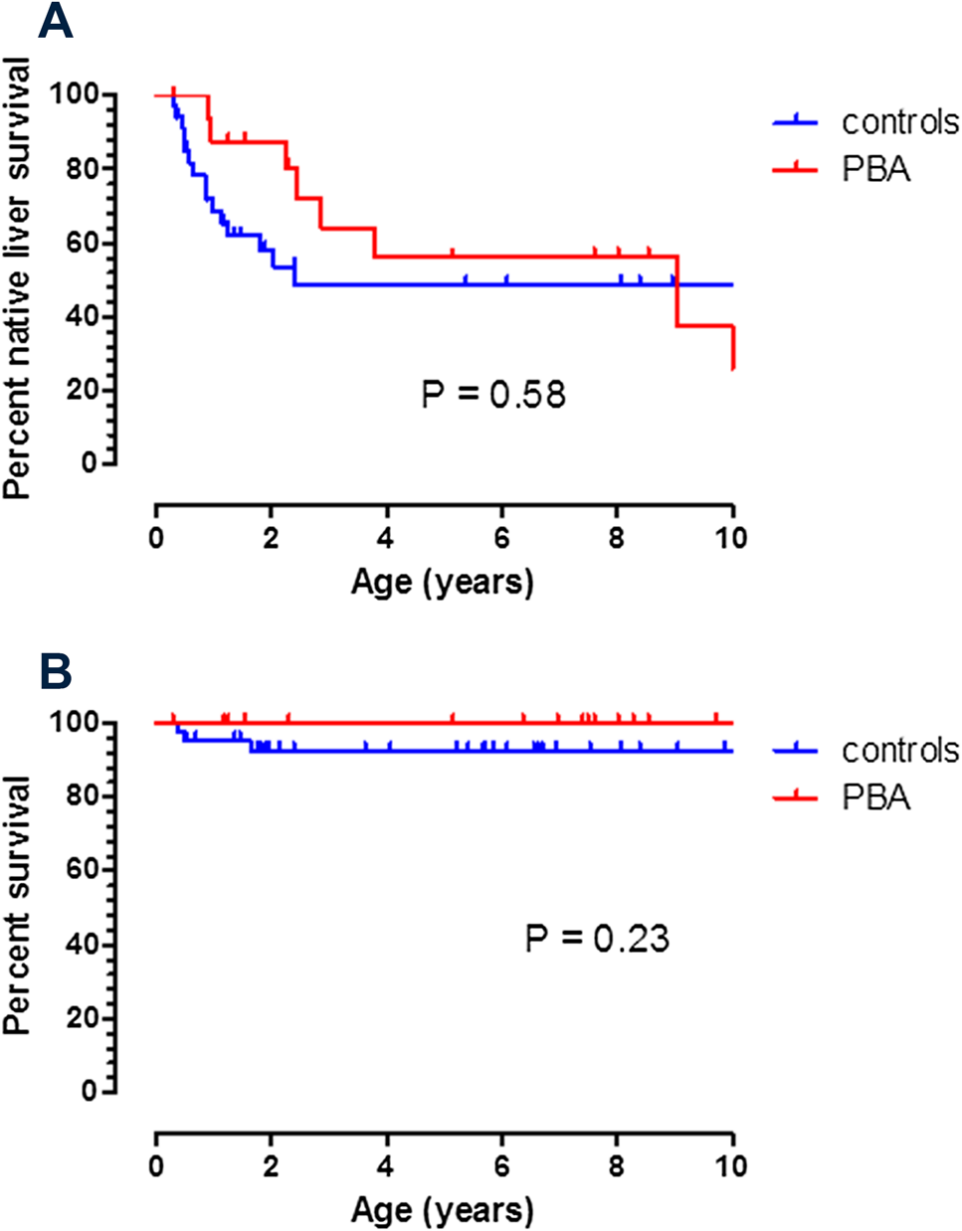
K–M survival curves in preterm biliary atresia and controls. **a** is native liver survival with death or transplant as end-points and **b** is actual survival

There was an increased but non-significant clearance of jaundice post-KPE in pre-term BA infants compared to controls [*n* = 12 (60%) vs. *n* = 20 (48%); *P* = 0.23]. Ultimate outcomes were death [*n* = 0 (0%) vs. *n* = 3 (7%)] and liver transplant [*n* = 12 (57%) + 1 primary transplant vs. *n* = 20 (48%); *P* = 0.58] in PBA vs. controls, respectively. Native liver survival post-KPE to 5 years (Fig. 4a) was unchanged between the two groups (56 vs. 49%; *P* = 0.58). Actual survival (Fig. 4b) at 5 years was again similar between the two groups (100 vs. 92%; *P* = 0.23). Median overall length of follow-up was 6.6 (0.51–28) years.

## Discussion

Biliary atresia is a rare disease with an incidence of 1 in 17,000 in the UK [10, 15]. The incidence of prematurity (< 37 weeks) is currently 7.1% of live births, which represents approximately 60,000 infants a year in the UK [16, 17]. The association of prematurity and biliary atresia is, therefore, possible by chance but obviously infrequent. There are three large-scale studies from Sweden [18], Taiwan [19] and, most recently, The Netherlands [20] in which the incidence of BA was reportedly higher in the premature population than in term infants with an odds ratio of 2.9 and 1.65 in the first two studies, respectively. Despite this reported increased incidence, outcomes and characteristics have been rarely reported, consisting largely of case reports (total reported = 5) [21–23]. Features such as diagnostic delay, single cases of twins, maternal morbidity and BASM have been mentioned as possible associations but not substantiated. In 2013, Chen et al. published the only other study reviewing outcomes of PBA infants in the literature with 27 cases from a Taiwanese national series [19]. Our cohort was characterized by an increased proportion of twinning which had not been identified in this Taiwanese study. They did identify a higher rate of other congenital malformations (18%); however BASM specifically was not found, although this is known to be rare in Asian series.

The twin pregnancy rate of 48% in our premature group is obviously striking. All of the PBA twins were discordant for BA in our series. In 1991, we reported an incidence of twins of 1.7% in an early series of 237 BA patients from Kings College Hospital, said to be consistent with the general population [24]. Our current series of almost 700 infants had 15 twins giving a rough incidence of about 2.1%. Clearly, twinning is a risk factor for prematurity which might simply explain the observation though there is a possibility that the presence of a twin may in itself be a risk factor for this disease. A recent review of the literature identified 30 discrete sets of twins with BA [21], with only two sets of dizygotic twins demonstrating concordance for the disease [25, 26]. Of the 7 reported monozygotic twins (MCDA) in the literature, all were discordant for the disease. Certainly within our series, the three MCDA twins were also discordant for BA. This may imply that an epigenetic factor is at play rather than being a purely genetic aetiology, possibly deranging the developing bile ducts.

Our PBA patients were older at diagnosis and operation compared to our controls. This was also found in the Taiwanese series; their KPE in the premature group occurred at a mean of 71 days, later than the term group by 19 days [19]. The prevalence of cholestatic jaundice in premature neonates is high with an incidence of nearly 25% in one large study [27]. Delayed initiation of enteral feeds, parenteral nutrition and recurrent sepsis all contribute to neonatal jaundice, with immaturity of bile acid metabolism and enterohepatic re-circulation contributing to the cholestatic picture [28]. It is likely that BA as a differential for persistent jaundice in the premature is initially ignored due to these confounding factors, which leads to a delayed diagnosis. The presence or absence of phototherapy seemed unconnected; however, use of parenteral nutrition was higher in those diagnosed > 50 days in our group, though not statistically so.

Interestingly, despite a relative delay to operation, our PBA group had a lower degree of cholestatic jaundice compared to term controls. Fibrosis at diagnosis was also lower as assessed directly by biopsy and using a validated surrogate (APRi) at the time of surgery. Although developed as a marker of fibrosis and hence prognosis following viral hepatitis [29], the Ishak score has since been adopted to quantify fibrosis in other pathologies, including biliary atresia [30]. The mean Ishak score was lower in the PBA group at diagnosis but this is equivalent to controls by the time of surgery. It seems unlikely but possible that the longer deferral time from diagnosis to surgery in the PBA group may account for the correction of this effect. The longer time to surgery in the PBA group is possibly accounted for by the higher frequency of co-morbidities resulting in the need for more thorough pre-operative preparation.

Against expectations, our outcomes were comparable, if not better than our control group. This was despite a longer time to KPE in the PBA cohort and a higher incidence of BASM, both of which are known poor prognostic factors [2, 31]. In contrast, there was poor clearance of jaundice in the Taiwanese study of only 37% (compared to 62% in their term cohort) with correspondingly worse native liver survival (at 18 months 50 vs. 73%) [19]. Similarly, the outcome in the Dutch series was poor with only 23% of their 28 infants clearing their jaundice to normal levels. The reason for the difference between our studies is unclear. Both series are national series derived from many centres with presumably variable experience whereas ours was from a single institution with an excellent published track record dating back to the 1980s. There was more heterogeneity in our premature patient group, as our reported BASM frequency was almost double than that of the Taiwanese group. Our previous comparative study suggested that those infants with BASM fare worse than that in ‘isolated BA’ groups [32]. One would, therefore, expect our outcomes to be worse, which did not seem to be the case. The Taiwanese group had a slightly older mean gestation (34 + 3 weeks) and their infants also took on average 7 days longer to KPE than our group. They concluded that the immature liver may be more sensitive to cholestatic injury. We did not find any real evidence of this (see later) though the reasons are speculative.

The question at which time point does the fibrosis and liver damage occur remains unanswered. It is difficult to know which factor is more important for comparison between the PBA and control groups; post-natal or post-conceptual (corrected gestational) age at surgery. When correcting for prematurity, the median age at operation was actually at 23 days in the PBA cohort compared to 56 days in the CBA infants. Could this in some way explain our non-statistically significant superior outcomes in the PBA group? In 2007, we postulated that the underlying cholestatic process and the initiation of liver fibrosis might be asynchronous. Biopsies in three infants in the first week of life showed very minimal fibrosis, suggesting the onset of fibrosis was from the time of birth, even in those infants who demonstrably have an abnormal obstructed biliary tract from the first weeks of gestation [11]. This premature BA group gave us the opportunity to explore this concept further. We hypothesized that correcting for prematurity would have no effect on the degree of fibrosis at surgery, with post-natal age being the most important factor; however, that was not the case. There was indeed good correlation of liver fibrosis scores (in both PBA and control groups) with both post-natal and with post-conceptual age, though some of the infants (predominantly in the PBA group) who were many weeks old had very little liver fibrosis for their post-natal age. This was reflected in the observation that both fibrosis and APRi scores were less in the PBA cohort compared to their controls born around term, implying some kind of “protection” of prematurity perhaps. A possible explanation is that the lack of early enteral feeding seen in some of our groups was in some way contributory.

In conclusion, PBA infants appear to have similar if not better outcomes compared to term infants in our cohort, despite delayed diagnosis and higher frequency of the syndromic form. The high incidence of discordant twins supports the theory that epigenetic modifications could contribute to the pathogenesis of BA, suggesting that further studies are warranted in this rare association. Although cholestatic jaundice is much more common in the premature population as a whole, the same principles of initiation of appropriate investigation should still apply, particularly if the associations we have highlighted (twins and BASM) are present. Delayed diagnosis was commonly seen in this sub-set and is avoidable as liver biopsy was discriminatory in all where it was done.
